# Relationship between circadian eating behavior (daily eating frequency and nighttime fasting duration) and cardiovascular mortality

**DOI:** 10.1186/s12966-023-01556-5

**Published:** 2024-02-26

**Authors:** Weilun Cheng, Xing Meng, Jian Gao, Wenbo Jiang, Xinyi Sun, Ying Li, Tianshu Han, Dandan Zhang, Wei Wei

**Affiliations:** 1 Department of General Surgery, the Second Affiliated Hospital of Harbin Medical University, 246 Xuefu Road, Harbin, 150001 China; 2 Department of Clinical Nutrition, the First Affiliated Hospital of Harbin Medical University, 199 Dazhi Street, Harbin, 150001 China; 3 Department of Nutrition and Food Hygiene, School of Public Health, the National Key Discipline, Harbin Medical University, 157 Baojian Road, Harbin, 150081 China; 4 Department of Cardiology, the First Affiliated Hospital of Harbin Medical University, 199 Dazhi Street, Harbin, 150001 China; 5 Key Laboratory of Precision Nutrition and Health, Ministry of Education, Harbin Medical University, 157 Baojian Road, Harbin, 150081 China; 6 Department of Gynecology and Obstetrics, the First Affiliated Hospital of Harbin Medical University, 199 Dazhi Street, Harbin, 150001 China; 7 Department of Pharmacology, College of Pharmacy Key Laboratory of Cardiovascular Research, Ministry of Education, Harbin Medical University, 157 Baojian Road, Harbin, 150081 China

**Keywords:** Daily eating frequency, Nighttime fasting duration, Long-term health impacts, Cardiovascular health

## Abstract

**Background:**

Knowledge regarding the health impacts of daily eating frequency (DEF) and nighttime fasting duration (NFD) on mortality is very limited.

**Objective:**

This study aimed to examine whether DEF and NFD are associated with CVD and all-cause mortality.

**Methods:**

This was a prospective cohort study of a nationally representative sample from the United States, including 30,464 adults who participated in the National Health and Nutrition Examination Survey 2003–2014. Using 24-h dietary recall, DEF was assessed by the number of eating episodes, and NFD was calculated by the first and last eating time across a day. Death information was obtained from the National Death Index up to 2019. Weighted Cox proportional hazards regression models were used to assess survival relationships of DEF and NFD with mortality.

**Results:**

During 307,686 person-years of follow-up, 4560 deaths occurred, including 1824 CVD cases. After adjustment for confounders, compared to DEF at 4–6 times, participants whose DEF was less than 3 times had greater CVD [hazard-ratio (HR) = 1.33, 95% confidence-interval (CI): 1.06–1.67] and all-cause (HR = 1.16, 95% CI: 1.01–1.33) mortality risks. Furthermore, compared to NFD of 10 to 11 h, participants whose NFD was shorter than 10 h had HRs of 1.30 (95% CI: 1.08–1.55) for CVD mortality and 1.23 (95% CI: 1.08–1.39) for all-cause mortality. NFD longer than 14 h was also related to CVD mortality (HR = 1.37, 95% CI: 1.12–1.67) and all-cause mortality (HR = 1.36, 95% CI: 1.19–1.54). Similar results for the association of NFD and DEF with heart-specific and stroke-specific mortality were observed.

**Conclusion:**

This study found that DEF less than 3 times and NFD shorter than 10 h or longer than 14 h were independently associated with greater cardiovascular and all-cause mortality.

**Supplementary Information:**

The online version contains supplementary material available at 10.1186/s12966-023-01556-5.

## Introduction

Cardiovascular disease (CVD), as the leading cause of death, is an important public health concern in the United States and worldwide. Over the past decades, dietary studies have mainly focused on the quantity and quality of dietary composition, and they successfully identified abundant dietary risk factors related to the development of CVD, forming the foundation of the American Heart Association’s Diet and Lifestyle Recommendations [1]. Recently, emerging studies have demonstrated that in addition to the quantity and quality of food, circadian eating behavior is critical for the well-being of an organism [2, 3]. A few studies have found that some circadian eating behaviors, such as breakfast skipping, late-night eating, high energy-intake at dinner, and eating time for specific food, are associated with morbidity and mortality of CVD [4–9]. However, few studies have examined whether daily eating frequency and nighttime fasting duration, as two major components of circadian eating behavior, are associated with the natural course of CVD.

Currently, evidence from prospective studies and randomized controlled trials (RCTs) has shown that less daily eating frequency is associated with higher levels of blood pressure and serum cholesterol and a greater incidence of diabetes, probably increasing the risk status of CVD [10]. However, eating behavior for less daily eating frequency is usually related to behavior for longer nighttime fasting duration, and recent RCTs have found that prolonged nighttime fasting duration can improve body weight, glucose, insulin resistance and inflammation [11–14]. Therefore, it is imperative to clarify the long-term health impact of daily eating frequency and nighttime fasting duration on cardiovascular mortality to add important knowledge in terms of circadian eating behaviors and long-term survival among free-living populations. To achieve this, we aimed to simultaneously investigate the association of daily eating frequency and nighttime fasting duration with cardiovascular and all-cause mortality in a nationally representative sample in the United States.

## Methods

### Study population

The National Health and Nutrition Examination Survey (NHANES) is a multistage stratified sampling study among the noninstitutionalized civilian population of the United States [15]. This study enrolled participants aged over 18 years who participated in NHANES from 2003 to 2014. After excluding participants who had missing information for food intake and mortality, 30,646 participants, including 15,072 men and 15,392 women, were included in this study. Institutional review board approval was obtained from the National Center for Health Statistics, and written informed consent was obtained before data collection.

### Exposure

Participants’ food intake on two nonconsecutive days was collected by two 24-h dietary recall interviews. The first 24-h dietary recall was conducted in person, and the second 24-h dietary recall was conducted 3–10 days afterward via telephone. During the interview, the participants were requested to report the intake time of each food and beverage. The number of eating episodes was defined as the number of time stamps associated with calorie-containing food or beverage consumption, and a conservative 50 kcal cutoff was used to define an eating episode [10]. The mean daily eating frequency was calculated by the average of two-day eating episodes. The mean daily nighttime fasting duration was calculated using the following algorithm: 24 h—last eating time + first eating time. For example, a participant whose first eating time was at 7:00 a.m. and whose last eating time was at 6:00 p.m. would have a nighttime fasting duration of 24–18 + 7 = 13 h of fasting.

### Main outcome

The main outcome variables were all-cause, CVD, heart disease-specific and cerebrovascular disease-specific mortality. Mortality status was determined using the National Death Index (NDI), which is a highly reliable and widely used resource to identify mortality status. The NDI provided ICD-10 codes for underlying cause of death and up to 20 contributing causes of death. Only matches with a high degree of certainty were accepted. The detailed information can be found at https://www.cdc.gov/nchs/data-linkage/mortality-public.htm. Death due to heart disease includes acute rheumatic fever and chronic rheumatic heart diseases (codes I00–I09), hypertensive heart disease (codes I11), hypertensive heart and renal disease (codes I13), ischemic heart diseases (codes I20–I25), and other heart diseases (codes I26–I51). Codes I60–I69 were defined as cerebrovascular disease (stroke) mortality. CVD mortality was defined as death due to heart disease or cerebrovascular disease.

### Assessment of confounders

Confounders included age (years), sex (men or women), race (non-Hispanic white/non-Hispanic black/Mexican American/other), education level (< 9th grade, 9th-11th grade, high school graduate, GED or equivalent, some college or Associate in Arts degree, or college graduate or above), annual family income (< $20,000, $20,000—$45,000, $45,000—$75,000, $75,000—$100,000, or > $100,000), regular exercise habits (yes/no), smoking (yes/no), drinking (yes/no), body mass index (BMI, kg/m^2^), sleep duration (hour), night shift work (yes/no), daily energy intake (kcal/d), alternative healthy eating index (AHEI), fasting plasma glucose (mmol/l), plasma triglycerides (TG, mmol/l), plasma total cholesterol (TC, mmol/l), drug use for controlling glucose/hypertension/dyslipidemia (yes/no), family history of CVD (yes/no), meal skipping (breakfast/lunch/dinner/none) and dietary data surveyed on weekends.

### Statistical analysis

All analyses were performed with incorporation of sample weights, stratification, and clusters to account for the complex survey design. Demographic characteristics, prevalence of diseases and anthropometric measurements are presented as the mean (95% confidence interval, CI) for continuous variables and proportion (95% CI) for categorical variables, which were compared by using general linear regression and logistic regression. Restricted cubic spline (RCS) was used to visualize the survival dose‒response relationship of daily eating frequency and nighttime fasting duration with all-cause mortality, total CVD, heart disease and stroke by setting 5 knots at the 5th, 25th, 50th, 75th, and 95th percentiles. Analysis of variance (ANOVA) was used to examine the linear or nonlinear relationship of the spline [16]. The daily eating frequency and nighttime fasting duration were categorized into quintiles. Weighted Cox proportional hazards (CPH) models were developed to evaluate the survival relationship of daily eating frequency and nighttime fasting duration with all-cause mortality, CVD, heart disease and stroke while accounting for the complex survey design. Survival time was months between the NHANES interview date and death due to CVD or the census date (31 December 2019).

We also controlled for a series of confounders, including age, sex, race, education level, annual family income, regular exercise habits, smoking, drinking, BMI, sleep duration, daily energy intake, AHEI, fasting plasma glucose, TG, TC, drug use for controlling glucose/hypertension/dyslipidemia, family history of CVD, meal skipping and dietary data surveyed on weekends in all weighted-CPH models. All statistical analyses were conducted by R 4.1.2, and two-sided *P* < 0.05 was considered to be statistically significant.

### Sensitivity analysis

Three sensitivity analyses were performed. The first analysis excluded the participants whose follow-up duration was less than 2 years to examine whether severe illness influences the results. The second analysis excluded participants who had breakfast, lunch or dinner skipping behaviors to examine whether these associations are influenced by meal skipping. The third analysis evaluated the interaction effects of daily eating frequency and nighttime fasting duration with potential confounders (age, sex, BMI, AHEI, smoking and drinking status) by respectively adding the interaction terms in the CPH models.

## Results

### Baseline characteristics

During a total of 307,686 person-years of follow-up (median follow-up 10.0 years, maximum follow-up 17.1 years), 4560 deaths, including 1824 deaths due to CVD, were documented. The baseline demographic, nutrition characteristics and CVD risk factors in terms of daily eating frequency by quintiles are presented in Table 1. Compared to daily eating frequency more than 3 times (quintile 2 to quintile 5), participants whose eating frequency was less than 3 times (quintile 1) were more likely to be younger, men and have a higher smoking rate, regular exercise habits, BMI, prevalence of diabetes, meal skipping rates, and a longer nighttime fasting duration with a later first eating time and earlier last eating time across a day. They also had a lower drinking rate, income, daily energy intake and AHEI (all *P* < 0.05).

**Table 1:** Differences for the baseline characteristics in terms of studying variables by quintiles of daily eating frequency

**Variables**	**Daily eating frequency**					*P*-value
	Quintile 1 (< 3 times, *N* = 8410)	Quintile 2 (3 to 3.5 times, *N* = 8380)	Quintile 3 (3.5 to 4 times, *N* = 3337)	Quintile 4 (4 to 4.5 times, *N* = 5485)	Quintile 5 (4.5 to 6 times, *N* = 4852)	
Age (years)	44.2 (0.36)	45.8 (0.35)	47.1 (0.41)	47.9 (0.34)	47.4 (0.37)	< 0.001
Men, %	51.8 (0.6)	50.2 (0.6)	48.5 (1.1)	45.9 (0.7)	45.8 (0.9)	< 0.001
Non-Hispanic white, %	59.8 (2.0)	68.0 (1.6)	72.2 (1.5)	74.5 (1.2)	77.9 (1.2)	< 0.001
Smoking, %	27.8 (0.8)	26.1 (0.8)	23.8 (1.0)	22.8 (0.7)	21.0 (0.8)	< 0.001
Drinking, %	63.5 (0.9)	69.4 (0.7)	71.1 (1.2)	73.3 (1.0)	75.9 (1.0)	< 0.001
College graduate or above, %	46.9 (1.0)	54.1 (1.0)	63.1 (1.3)	63.1 (1.3)	69.7 (1.2)	< 0.001
Household income over $75,000, %	8.7 (0.6)	12.5 (0.9)	16.2 (1.1)	18.7 (1.0)	22.9 (1.4)	< 0.001
Exercised regularly, %	49.9 (0.7)	44.4 (0.8)	40.2 (1.1)	37.8 (1.2)	34.2 (1.2)	< 0.001
Sleep duration (hours)	6.9 (0.02)	6.9 (0.02)	7.0 (0.02)	6.9 (0.02)	6.9 (0.02)	0.440
Night shift work,%	0.6 (0.1)	0.6 (0.1)	0.2 (0.1)	0.3 (0.1)	0.4 (0.1)	< 0.001
Daily energy intake (kcal/d)	1885.4(12.6)	2090.9(14.3)	2160.0 (20.0)	2230.3 (12.9)	2413.5 (16.9)	< 0.001
AHEI	47.5 (0.18)	49.0 (0.19)	50.3 (0.26)	50.9 (0.19)	52.1 (0.21)	< 0.001
Meal skipping, %						
Breakfast skipping	11.4 (0.6)	5.4 (0.3)	2.3 (0.4)	2.5 (0.3)	2.2 (0.2)	< 0.001
Lunch skipping	14.3 (0.6)	8.2 (0.4)	6.6 (0.6)	6.7 (0.4)	6.1 (0.5)	< 0.001
Dinner skipping	2.8 (0.2)	1.9 (0.2)	1.0 (0.2)	1.2 (0.2)	1.4 (0.2)	< 0.001
Nighttime fasting duration (hours)	12.5 (0.04)	11.7 (0.04)	11.2 (0.04)	10.8 (0.04)	10.2 (0.04)	< 0.001
First eaten time	8:09 (1.8)	7:57 (2.4)	7:46 (2.4)	7:33 (1.8)	7:42 (1.8)	< 0.001
Last eaten time	20:09 (1.2)	20:21 (1.8)	20:31 (1.2)	20:36 (1.8)	20:46 (1.2)	< 0.001
BMI, kg/m^2^	29.0(0.10)	28.8 (0.09)	28.8 (0.18)	28.4 (0.15)	28.0 (0.13)	< 0.001
Hypertension, %	36.0 (0.8)	37.0 (0.8)	37.0 (1.1)	37.6 (0.9)	34.7 (0.9)	< 0.001
SBP, mm Hg	122.4 (0.3)	122.1 (0.3)	121.7 (0.4)	121.3 (0.3)	120.9 (0.3)	< 0.001
DBP, mm Hg	70.0 (0.2)	70.1 (0.2)	70.4 (0.3)	70.2 (0.3)	70.9 (0.3)	0.005
Diabetes, %	12.2 (0.4)	11.5 (0.4)	12.4 (0.9)	10.8 (0.5)	9.9 (0.7)	< 0.001
Fasting Glucose, mmol/l	5.86 (0.04)	5.85 (0.04)	5.76 (0.05)	5.77 (0.04)	5.68 (0.04)	< 0.001
Dyslipidemia, %	22.5 (0.7)	27.0 (0.5)	11.6 (0.4)	20.4 (0.5)	18.5 (0.5)	< 0.001
TC, mmol/l	5.00 (0.01)	5.05(0.02)	5.04 (0.03)	5.10 (0.02)	5.09 (0.02)	0.212
LDL-C, mmol/l	2.94 (0.02)	2.97 (0.02)	2.93 (0.03)	3.02 (0.02)	2.95 (0.03)	0.262
HDL-C, mmol/l	1.33 (0.01)	1.35 (0.01)	1.37 (0.01)	1.40 (0.01)	1.43 (0.01)	0.013
TG, mmol/l	1.49 (0.02)	1.54 (0.02)	1.49 (0.04)	1.52 (0.04)	1.43 (0.03)	0.262

*BMI* body mass index, *AHEI* alternative healthy eating index, *TC* plasma total cholesterol, *TG* plasma triglycerides

Continuous variables were presented as mean (Standard error), and categorical variables were presented as percentage (Standard error)

General linear models adjusting for age and sex, and chi-square tests were used to compare baseline characteristics as a function of fasting duration by different groups. All analyses were based on estimates with sample weights

The baseline demographic, nutrition characteristics and CVD risk factors in terms of nighttime fasting duration by quintiles are presented in Table 2. Compared to participants with a nighttime fasting duration of more than 10 h (quintile 2 to quintile 5), participants whose nighttime fasting duration was less than 10 h (quintile 1) were more likely to be men and non-Hispanic white. They had higher smoking and drinking rates, education levels, household income and daily energy intake, as well as a higher daily eating frequency, with an earlier first eating time and later last eating time across a day (all *P* < 0.05). Furthermore, compared to nighttime fasting duration less than 14 h (quintile 1 to quintile 4), participants whose nighttime fasting duration was more than 14 h (quintile 5) had lower levels of daily energy intake and AHEI, higher rates of meal skipping, and less eating frequency, with later first eating time and earlier last eating time across a day (all *P* < 0.05).

**Table 2:** Differences for the baseline characteristics in terms of studying variables by quintiles of nighttime fasting duration

**Variables**	**Nighttime fasting duration**					*P*-value
	Quintile 1 (≤ 10 h, *N* = 7896)	Quintile 2 (10 to 11 h, *N* = 5116)	Quintile 3 (11 to 12 h, *N* = 5480)	Quintile 4 (12 to 14 h, *N* = 6505)	Quintile 5 (≥ 14 h, *N* = 5467)	
Age (years)	46.8 (0.32)	47.9 (0.32)	47.3 (0.32)	45.9 (0.39)	42.8 (0.42)	< 0.001
Men, %	55.0 (0.7)	50.3 (0.9)	45.7 (0.8)	43.8 (0.8)	44.9 (0.8)	< 0.001
Non-Hispanic white, %	74.2 (1.3)	73.7 (1.3)	70.6 (1.6)	66.9 (1.5)	59.1 (2.0)	< 0.001
Smoking, %	28.5 (0.8)	23.0 (0.7)	22.9 (0.8)	21.3 (0.8)	25.7 (0.8)	< 0.001
Drinking, %	74.3 (0.8)	72.8 (1.1)	70.8 (1.0)	67.1 (0.9)	62.7 (1.0)	< 0.001
College graduate or above, %	30.4 (0.6)	19.6 (0.4)	18.6 (0.4)	19.4 (0.4)	12.1 (0.4)	< 0.001
Household income over $75,000, %	17.4 (1.1)	17.4 (1.0)	16.1 (1.0)	13.9 (1.0)	8.9 (0.7)	< 0.001
Exercised regularly, %	39.6 (1.1)	40.1 (1.1)	41.4 (1.0)	43.0 (1.0)	47.9 (0.9)	< 0.001
Sleep duration (hours)	6.7 (0.02)	6.9 (0.02)	7.0 (0.02)	7.1 (0.02)	7.0 (0.02)	< 0.001
Night shift work,%	0.7 (0.1)	0.2 (0.1)	0.1 (0.1)	0.3 (0.1)	0.9 (0.1)	< 0.001
Daily energy intake (kcal/d)	2365.8 (14.3)	2200.5 (16.0)	2094.5 (12.5)	2006.7 (13.9)	1868.9 (13.2)	< 0.001
AHEI	49.5 (0.19)	50.5 (0.20)	50.3 (0.19)	50.2 (0.20)	48.4 (0.19)	< 0.001
Meal skipping, %						< 0.001
Breakfast skipping	3.9 (0.3)	2.3 (0.3)	2.3 (0.3)	4.2 (0.3)	16.5 (0.7)	< 0.001
Lunch skipping	8.5 (0.4)	6.6 (0.5)	8.8 (0.6)	8.1 (0.4)	12.6 (0.6)	< 0.001
Dinner skipping	1.5 (0.1)	1.3 (0.2)	1.5 (0.2)	1.7 (0.2)	3.2 (0.3)	< 0.001
Daily eating frequency	4.4 (0.02)	4.0 (0.02)	3.8 (0.02)	3.6 (0.02)	3.0 (0.02)	< 0.001
First eaten time	6:40 (1.2)	7:41 (1.8)	8:00 (1.8)	8:21 (1.8)	9:10 (2.4)	< 0.001
Last eaten time	21:00 (1.2)	20:39 (1.2)	20:25 (1.8)	20:06 (1.2)	19:42 (0.03)	< 0.001
BMI, kg/m^2^	28.4 (0.11)	28.6 (0.13)	28.5 (0.12)	28.6 (0.11)	28.9 (0.14)	< 0.001
Hypertension, %	37.0 (0.7)	38.0 (0.9)	36.7 (0.9)	36.4 (1.0)	33.1 (0.9)	< 0.001
SBP, mm Hg	121.5 (0.25)	122.0 (0.36)	121.8 (0.33)	121.9 (0.31)	121.3 (0.34)	0.066
DBP, mm Hg	70.6 (0.21)	70.9 (0.28)	70.2 (0.26)	70.1 (0.28)	69.1 (0.28)	< 0.001
Diabetes, %	10.6 (0.6)	12.2 (0.6)	11.7 (0.5)	11.3 (0.5)	11.4 (0.6)	0.001
Fasting Glucose, mmol/l	5.79 (0.04)	5.80 (0.04)	5.81 (0.05)	5.78 (0.34)	5.81 (0.05)	0.040
Dyslipidemia, %	47.0 (0.7)	47.5 (0.8)	47.4 (0.9)	46.4 (0.9)	43.1 (1.2)	< 0.001
TC, mmol/l	5.05 (0.02)	5.09 (0.02)	5.08 (0.02)	5.02 (0.02)	5.01 (0.02)	0.248
LDL-C, mmol/l	2.96 (0.02)	3.01 (0.02)	2.98 (0.02)	2.95 (0.02)	2.91 (0.02)	0.305
HDL-C, mmol/l	1.37 (0.01)	1.39 (0.01)	1.38 (0.01)	1.37 (0.01)	1.34 (0.01)	< 0.001
TG, mmol/l	1.45 (0.02)	1.48 (0.03)	1.52 (0.03)	1.51 (0.02)	1.58 (0.04)	< 0.001

*BMI* body mass index, *AHEI* alternative healthy eating index, *TC* plasma total cholesterol, *TG* plasma triglycerides

Continuous variables were presented as mean (Standard error), and categorical variables were presented as percentage (Standard error)

General linear models adjusting for age and sex, and chi-square tests were used to compare baseline characteristics as a function of fasting duration by different groups. All analyses were based on estimates with sample weights

### Dose‒response relationship of daily eating frequency and nighttime fasting duration with mortality

**Fig. 1:**
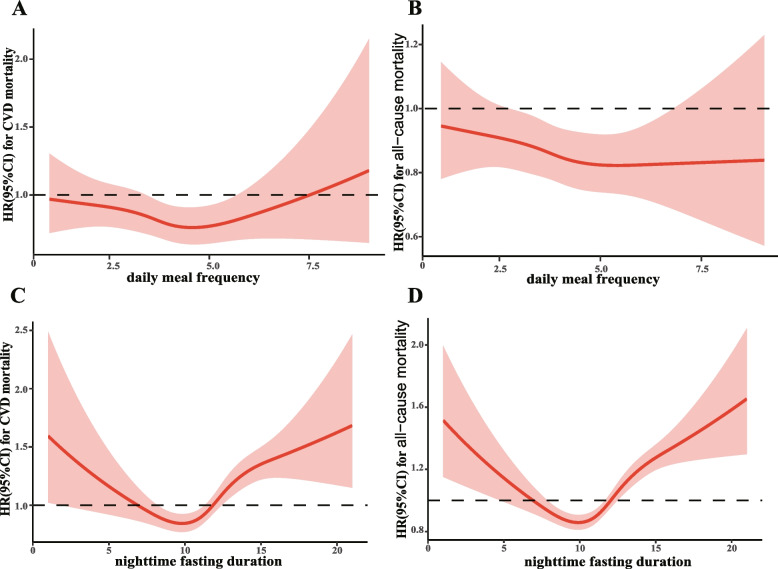
Dose–response association for the Daily eating frequency and nighttime fasting duration and mortalities for heart-disease specific and stroke specific. Legends: **A** daily eating frequency and CVD mortality; **B** daily eating frequency and all-cause mortality; **C** nighttime fasting duration and CVD mortality; **D** nighttime fasting duration and all-cause mortality

Daily eating frequency was significantly and negatively associated with nighttime fasting duration (*r* = -0.54, *P* < 0.001; Supplementary Fig. 1). We first visualized the dose‒response relationship of daily eating frequency and nighttime fasting duration with CVD and all-cause mortality. The mortality risk of CVD persistently decreased with increasing daily eating frequency, and the lowest risk point was reached when the daily eating frequency was approximately 4.5 times, after which it gradually increased. Similarly, the all-cause mortality risk persistently decreased with increasing daily eating frequency, and the lowest risk point was reached when the daily eating frequency was approximately 5.5 times (Fig. 1-A and B). For nighttime fasting duration, the mortality risks of CVD and all-cause CVD gradually decreased with the nighttime fasting duration increased to 10–11 h, and then the mortality risk gradually increased with increased nighttime fasting duration, which all showed strong U-shaped relationships (all P _for non-linearity_ < 0.01) (Fig. 1-C and D). A similar dose‒response varying trend between daily eating frequency and heart disease-specific and stroke-specific mortality was also observed (Supplementary-Fig. 2A and B), with the nighttime fasting duration still having U-shaped relationships with heart disease-specific and stroke-specific mortality (Supplementary Fig. 2C and D).

### Hazards ratio (HR) and 95% CI for association of daily eating frequency and nighttime fasting duration with mortality

Based on the dose‒response relationship between daily eating frequency and mortality, we set the highest quintile of daily eating frequency (4.5–6 times per day) as the reference group in the weight CPH models. As indicated by weighted HRs and 95% CIs, compared with those in the highest quintile 1, participants in the lowest quintile of daily eating frequency (< 3 times per day) had greater mortality risks of CVD and all-cause mortality in Model 2 with adjustment for demographic and nutrition characteristics and risk factors for CVD (HR: 1.39, 95% confidence interval [95% CI]: 1.12 to 1.71 for CVD mortality; HR = 1.23, 95% CI: 1.07 to 1.40 for all-cause mortality) (Table 3). A similar significant result was also observed for daily eating frequency and heart disease-specific mortality (HR = 1.51, 95% CI: 1.17 to 1.94) but not stroke-specific mortality (HR = 0.89, 95% CI: 0.54 to 1.48) in Model 2, as shown in Table 3.

**Table 3:** Association of daily eating frequency with mortalities of all-cause, CVD, heart disease and stroke

	Daily eating Frequency				
	Quintile 1 (< 3 times)	Quintile 2 (3 to 3.5 times)	Quintile 3 (3.5 to 4 times)	Quintile 4 (4 to 4.5 times)	Quintile 5 (4.5 to 6 times)
All-cause mortality					
Death/person-years	1879/108202	759/49470	789/54242	495/35711	635/59486
Unadjusted	1.67 (1.48–1.89)	1.54 (1.34–1.77)	1.39 (1.21–1.59)	1.44 (1.25–1.66)	1 (ref.)
Model 1	1.38 (1.21–1.56)	1.30 (1.13–1.49)	1.22 (1.06–1.42)	1.23 (1.06–1.42)	1 (ref.)
Model 2	1.23 (1.07–1.40)	1.23 (1.07–1.41)	1.16(0.99–1.34)	1.21(1.04–1.39)	1 (ref.)
Model 3	1.16 (1.01–1.33)	1.20(1.05–1.37)	1.14(0.98–1.33)	1.21(1.04–1.39)	1 (ref.)
CVD mortality					
Death/person-years	797/108202	297/49470	319/54242	181/35711	229/59486
Unadjusted	2.12 (1.75–2.57)	1.79 (1.44–2.21)	1.72(1.40–2.10)	1.49(1.18–1.88)	1 (ref.)
Model 1	1.63 (1.33–2.00)	1.41 (1.14–1.75)	1.44 (1.17–1.77)	1.22(0.95–1.56)	1 (ref.)
Model 2	1.39 (1.12–1.71)	1.28 (1.04–1.59)	1.33 (1.08–1.63)	1.78 (0.92–1.50)	1 (ref.)
Model 3	1.33 (1.06–1.67)	1.27 (1.02–1.58)	1.32 (1.07–1.63)	1.18 (0.92–1.51)	1 (ref.)
Heart disease-specific mortality					
Death/person-years	512/108202	194/49470	219/54242	113/35711	143/59486
Unadjusted	2.35 (1.86–2.98)	2.06 (1.57–2.69)	2.07 (1.62–2.64)	1.64 (1.24–2.18)	1 (ref.)
Model 1	1.87 (1.47–2.39)	1.64 (1.24–2.15)	1.80 (1.41–2.30)	1.36 (1.03–1.81)	1 (ref.)
Model 2	1.51 (1.17–1.94)	1.44 (1.10–1.89)	1.62 (1.27–2.08)	1.29 (0.98–1.71)	1 (ref.)
Model 3	1.44 (1.09–1.90)	1.43 (1.08–1.90)	1.62 (1.25–2.10)	1.30 (0.98–1.74)	1 (ref.)
Stroke-specific mortality					
Death/person-years	113/108202	32/49470	45/54242	28/35711	34/59486
Unadjusted	0.72 (0.48–1.08)	0.77 (0.48–1.21)	1.10 (0.67–1.82)	1.28 (0.94–1.74)	1 (ref.)
Model 1	0.77 (0.50–1.21)	0.76 (0.47–1.23)	1.10 (0.68–1.77)	1.30 (0.94–1.81)	1 (ref.)
Model 2	0.89 (0.54–1.48)	0.85 (0.52–1.37)	1.19 (0.73–1.96)	1.39 (0.99–1.91)	1 (ref.)
Model 3	0.90 (0.52–1.57)	0.89 (0.51–1.57)	1.28 (0.76–2.17)	1.47 (1.04–2.07)	1 (ref.)

Data is weighted HR and 95%CI

Model 1 is unadjusted model with additionally adjusted for age, age, sex, race, education level, annual family income, regular exercise habitus, smoking, drinking, BMI, sleep duration, daily energy intake, AHEI, meal skipping and whether dietary data was surveyed on weekend

Model 2 is model 1 with additionally adjusted for fasting plasma glucose, TG, TC, drug use for controlling glucose/ hypertension/ dyslipidemia, family history of CVD

Model 3 is model 2 with additionally adjusted for nighttime fasting duration

Furthermore, based on the U-shaped relationships between nighttime fasting duration and mortality, the second quintile of nighttime fasting duration was set as the reference group in the weight CPH models. As indicated by weighted HRs and 95% CIs, compared with those in quintile 2, participants in the lowest quintile (quintile 1) were more likely to die due to CVD (HR = 1.21, 95% CI: 1.01 to 1.44) and all-cause CVD (HR = 1.16, 95% CI: 1.03 to 1.32) in Model 2 with adjustment for demographic, nutrition characteristics and risk factors for CVD (Table 2). Similarly, participants in the highest quintile (quintile 5) had higher mortality risk of CVD mortality (HR: 1.37, 95% CI: 1.12 to 1.67) and all-cause mortality (HR: 1.36, 95% CI: 1.19 to 1.54). A similar significant result was also observed in terms of nighttime fasting duration and heart disease-specific mortality (HR = 1.30, 95% CI: 1.02 to 1.66 for quintile 1; HR = 1.72, 95% CI: 1.34 to 2.19 for quintile 5) and stroke-specific mortality (HR = 1.79, 95% CI: 1.04 to 3.09 for quintile 1; HR = 1.81, 95% CI: 1.09 to 2.99 for quintile 5) in Model 2, as shown in Table 4.

**Table 4:** Association of nighttime fasting duration with mortalities of all-cause, CVD, heart disease and stroke

	Nighttime fasting duration				
	Quintile 1 (≤ 10 h)	Quintile 2 (10 to 11 h)	Quintile 3 (11 to 12 h)	Quintile 4 (12 to 14 h)	Quintile 5 (≥ 14 h)
All-cause mortality					
Death/person-years	1021/78644	673/51160	808/54800	1066/65050	992/55763
Unadjusted	1.07 (0.94–1.22)	1 (ref.)	1.18 (1.03–1.35)	1.23 (1.08–1.41)	1.38 (1.22–1.58)
Model 1	1.15 (1.01–1.31)	1 (ref.)	1.15 (1.01–1.32)	1.27 (1.13–1.43)	1.57 (1.39–1.78)
Model 2	1.16 (1.03–1.32)	1 (ref.)	1.13 (0.99–1.30)	1.23 (1.10–1.39)	1.47 (1.29–1.68)
Model 3	1.23 (1.08–1.39)	1 (ref.)	1.10 (0.96–1.26)	1.16 (1.04–1.30)	1.36 (1.19–1.54)
CVD mortality					
Death/person-years	388/78644	260/51160	323/54800	433/65050	420/55763
Unadjusted	1.10 (0.91–1.32)	1 (ref.)	1.25 (1.02–1.54)	1.30 (1.06–1.59)	1.56 (1.30–1.86)
Model 1	1.19 (0.99–1.42)	1 (ref.)	1.23 (1.01–1.49)	1.30 (1.06–1.59)	1.68 (1.42–1.99)
Model 2	1.21 (1.01–1.44)	1 (ref.)	1.19 (0.98–1.44)	1.23 (1.00–1.52)	1.52 (1.28–1.80)
Model 3	1.30 (1.08–1.55)	1 (ref.)	1.15 (0.95–1.39)	1.14 (0.93–1.40)	1.37 (1.12–1.67)
Heart disease-specific mortality					
Death/person-years	255/78644	165/51160	206/54800	276/65050	280/55763
Unadjusted	1.19 (0.93–1.51)	1 (ref.)	1.32 (1.02–1.72)	1.35 (1.07–1.70)	1.77 (1.41–2.23)
Model 1	1.27 (1.00–1.62)	1 (ref.)	1.27 (0.99–1.64)	1.35 (1.07–1.70)	1.95 (1.55–2.47)
Model 2	1.30 (1.02–1.66)	1 (ref.)	1.23 (0.95–1.58)	1.26 (1.00–1.59)	1.72 (1.34–2.19)
Model 3	1.41 (1.11–1.79)	1 (ref.)	1.18 (0.91–1.52)	1.15 (0.91–1.44)	1.48 (1.12–1.97)
Stroke-specific mortality					
Death/person-years	57/78644	31/51160	43/54800	66/65050	55/55763
Unadjusted	1.54 (0.95–2.52)	1 (ref.)	1.52 (0.86–2.68)	1.80 (1.06–3.03)	1.77 (1.12–2.79)
Model 1	1.76 (1.02–3.05)	1 (ref.)	1.49 (0.80–2.76)	1.73 (0.99–3.04)	1.97 (1.22–3.19)
Model 2	1.79 (1.04–3.09)	1 (ref.)	1.44 (0.78–2.68)	1.65 (0.92–2.98)	1.81 (1.09–2.99)
Model 3	1.89 (1.11–3.23)	1 (ref.)	1.40 (0.75–2.63)	1.59 (0.84–2.98)	1.84 (1.04–3.24)

Data is weighted HR and 95%CI

Model 1 is unadjusted model with additionally adjusted for age, age, sex, race, education level, annual family income, regular exercise habitus, smoking, drinking, BMI, sleep duration, daily energy intake, AHEI, meal skipping and whether dietary data was surveyed on weekend

Model 2 is model 1 with additionally adjusted for fasting plasma glucose, TG, TC, drug use for controlling glucose/ hypertension/ dyslipidemia, family history of CVD

Model 3 is model 2 with additionally adjusted for daily eating frequency

After the daily eating frequency and nighttime fasting duration were included in the fully adjusted model, daily eating frequency less than 3 times was still associated with greater mortality risks of all-cause (HR: 1.16, 95% CI: 1.01 to 1.33), CVD (HR: 1.33, 95% CI: 1.06 to 1.67) and heart disease (HR: 1.44, 95% CI: 1.09 to 1.90), as shown in Table 3, in the fully adjusted model. Moreover, nighttime fasting duration less than 10 h or more than 14 h was significantly associated with greater mortality risks of all-cause (HR: 1.23, 95% CI: 1.08 to 1.39 for quintile 1; HR: 1.36, 95% CI: 1.19 to 1.54 for quintile 5), CVD (HR: 1.30, 95% CI: 1.08 to 1.55 for quintile 1; HR: 1.37, 95% CI: 1.12 to 1.67 for quintile 5), heart disease-specific (HR: 1.41, 95% CI: 1.11 to 1.79 for quintile 1; HR: 1.48, 95% CI: 1.12 to 1.97 for quintile 5) and stroke-specific (HR: 1.89, 95% CI: 1.11 to 3.23 for quintile 1; HR: 1.84, 95% CI: 1.04 to 3.24 for quintile 5), as shown in Table 4, in the fully adjusted model.

### Sensitivity analysis

The first sensitivity analysis showed that excluding participants whose follow-up duration was less than 24 months yielded similar results for both CVD mortality and all-cause mortality (Supplementary Tables 1 and 2), suggesting that serious illness likely would not influence the above results. The second sensitivity analysis excluded participants with breakfast, lunch or dinner skipping, and the results of the weight CPH model were similar to those of the total sample (Supplementary Tables 3, 4, 5, 6, 7 and 8). The third sensitivity analysis showed that age, sex, BMI, smoking and drinking status did not modify the association of daily eating frequency and nighttime fasting duration with all-cause mortality and CVD (Supplementary Table 9). However, the AHIE modified the association of daily eating frequency with the mortality risk of all-cause and CVD (P for interaction < 0.001). Therefore, we further examined the association of daily eating frequency with all-cause and CVD by the median value of the AHEI. The results indicated that the above association was more obvious for participants whose AHEI value was over the median but that it decreased among participants whose AHEI value was below the median.

## Discussion

To the best of our knowledge, this study represents the first instance of prospective evidence regarding the relationship between daily eating frequency and nighttime fasting duration with cardiovascular disease (CVD) and all-cause mortality. We conducted this research using a nationally representative sample from the United States, with a median follow-up period of 10.1 years. Our findings indicate that both daily eating frequency less than 3 times and nighttime fasting duration less than 10 h or longer than 14 h are independently associated with a greater risk of both CVD and all-cause mortality. Importantly, these associations remained significant even after accounting for classical known nutritional and cardiovascular risk factors.

The findings of this study, which reveal an increased risk of cardiovascular disease (CVD) and all-cause mortality associated with a daily eating frequency of less than three times per day, are consistent with research studies. For instance, a prospective analysis involving men showed that individuals who consumed meals only 1 to 2 times a day had a 26% higher risk of diabetes compared to those with a regular three-meal-a-day pattern [17]. Additionally, an intervention trial conducted without caloric restriction demonstrated that individuals who ate just one meal per day had 19% and 25% higher levels of total and LDL cholesterol, respectively, as well as elevated systolic and diastolic blood pressure, compared with participants whose meal frequency was 4 to 6 times a day [18]. Furthermore, irregular eating behaviors might contribute to the observed association. In this study, it was noted that participants with a daily eating frequency of less than three times were more likely to exhibit irregular eating habits, including meal skipping. A recent prospective study found that irregular meal patterns were associated with a higher incidence of CVD [19], and several cross-sectional studies have reported that individuals with irregular meal patterns were more prone to obesity, metabolic syndrome, dyslipidemia, and oxidative stress [20–22]. Intervention studies have also shown that individuals with irregular meal frequencies (3–9 eating occasions per day) had higher levels of insulin resistance and hyperlipidemia than those with six regular eating episodes per day [23, 24]. Additionally, our data indicate that maintaining a consistent daily eating pattern of 4–6 times a day holds greater significance among participants who already have a high-quality daily diet than among those with low dietary quality. This observation implies that even individuals with generally healthy diets should pay close attention to both the frequency and regularity of their daily eating habits to reduce the risks of mortality associated with CVD.

In addition to daily eating frequency, the duration of nighttime fasting is another crucial aspect of circadian eating behavior. While current research in both animals and humans has consistently reported positive health effects associated with extended nighttime fasting, such as weight loss and improved glucose and lipid regulation [11–14], our study has uncovered a more complex relationship. Our findings indicate that the health impacts of nighttime fasting duration are not straightforward. Specifically, individuals with a nighttime fasting duration of less than 10 h or more than 14 h both exhibited greater mortality risks related to total CVD, heart disease, and stroke. These findings regarding the health impacts of shortened nighttime fasting duration to some extent align with previous studies. Previous cross-sectional studies have consistently reported that individuals who habitually engage in late-night eating exhibit a higher prevalence of obesity, diabetes, and metabolic syndrome, accompanied by elevated levels of inflammation [25–28]. Additionally, a prospective study revealed that men who consumed nighttime meals had a greater incidence of coronary heart disease than those who refrained from eating during the night [29]. This association may be linked to disruptions in rhythmic secretion of insulin, which might be a key mechanism underlying these observations. Under normal circumstances, insulin secretion follows a rhythmic pattern characterized by a gradual increase during the day and a decrease during the night [30]. Late-night eating can directly stimulate insulin secretion, leading to disruption of this natural rhythmic pattern [31]. Both in vivo and in vitro experiments have demonstrated that mistimed insulin signaling can disturb the circadian organization of mouse behavior and expression of clock genes [32]. This disruption occurs through increased synthesis of the PERIOD protein via insulin and IGF-1 receptor signaling, ultimately resulting in metabolic disorder [32].

Furthermore, the most important finding in this study pertains to the observation that an extended nighttime fasting duration is linked to a higher mortality risk, particularly in relation to stroke-specific mortality. It is worth noting that several studies have previously underscored the positive health effects associated with nighttime fasting durations exceeding 14 h [11–14]. However, it is important to consider the context in which these studies were conducted. Many of the studies emphasizing the benefits of prolonged nighttime fasting were carried out among individuals who were overweight or had obesity or prediabetes [11–13]. These investigations often had specific goals, such as weight loss or glucose control, and typically had professional staff guidance during intervention [11–13]. These factors may contribute to the disparities in results between our study and previous RCTs. Although long-term observation evidence based on the general population focusing on the health impacts of daily fasting duration on CVD is relatively limited, the findings may be supported by Ramadan fasting. Ramadan fasting requires participants to maintain a fasting duration ranging from 14 to 20 h per day, and previous studies have found that the incidence of stroke increases among Bedouin Arabs during Ramadan [33, 34]. Moreover, it has been reported that meal skipping or prolonged fasting duration is associated with migraine [35], and the incidence of migraine has been documented to be associated with a higher incidence of stroke and heart disease [36]. Mechanistically, it has been reported that expression of more than 3000 genes has a 12-h biological rhythm for maintaining homeostasis of various hormones, including ghrelin, cortisol, melatonin, leptin, and serotonin [37–42]. A few studies have demonstrated that prolonged nighttime fasting duration elevates circulating ghrelin levels at night [43, 44], probably disrupting the circadian pattern of hormone homeostasis, which may increase the mortality risk of CVD. Additionally, temporal fasting has been found to have a significant impact on the immune system. Specifically, it can lead to a drastic reduction in levels of B cells in Peyer's patches, with germinal center B cells undergoing apoptosis. This effect on the immune system has the potential to impair its normal function, which may have implications for overall health and potentially contribute to adverse health outcomes [45].

### Strength and limitations

The major strengths of this population-based study are the use of a nationally representative sample with high-quality dietary data, strengthening the generalization of our findings to the free-living population in the United States. Moreover, the follow-up duration was up to 17 years, and the rates of matched records in NHANES 2003–2014 Linked Mortality File were relatively high. Last, the association documented in this study is relatively robust with adjustment for a variety of classic confounders of CVD, including dietary and biochemical risk factors. However, we also recognize that the study has certain limitations. First, although self-reported 24-h dietary recall is the most valid and widely used method to collect diet information in nutritional epidemiological studies, it has subjective measurement error due to day-to-day variations in food intake. Second, consecutive days of 24-h dietary information collection were not available. Therefore, last and first eating times were used to estimate the fasting duration, making the true estimation of the fasting period from one night to the next morning impossible. This probably led to residual confounding, though confounders related to fasting duration, including night-shift workers, sleep duration and weekend dietary reports, were adjusted. Third, we controlled for a series of potential confounders; however, this study was observational in nature, and other unmeasured confounding factors cannot be ruled out.

### Clinical perspectives

Nutritional therapy is a critical element of CVD prevention and treatment. This study found that in addition to the quantity and quality of dietary factors, lowered eating frequency with meal skipping and shortened or prolonged fasting duration were all independently associated with total CVD and heart disease-specific and stroke-specific mortality, suggesting that circadian eating behaviors play important roles in the development of CVD. The guidelines and recommendations of the World Heart Federation in terms of dietary management in CVD should integrate and emphasize the importance of circadian eating behaviors. Cardiovascular professionals should be aware of the current findings from this study regarding the potential beneficial effects of regular meal patterns with daily eating frequencies ranging from 4 to 6 times and nighttime fasting durations ranging from 10 to 12 h on cardiovascular health. This information is of importance in providing individualized nutritional prevention and treatment plans for free-living populations. Moreover, this study was observational in nature, and in the future, the investigation should focus on the role of circadian eating behaviors on health through RCTs. Also, the mechanisms underlying the circadian eating behaviors and their relationship with health should be elucidated by animal experiments. This will provide a more robust scientific basis for the updating and revision of nutritional disease prevention and treatment guidelines.

## Conclusion

This study found that a daily eating frequency less than 3 times and a nighttime fasting duration shorter than 10 h or longer than 14 h were independently associated with greater all-cause, CVD, heart disease-specific and stroke-specific mortality. Participants should maintain daily eating frequency from 4 to 6 times and maintain nighttime fasting duration at 11–12 h to reduce their mortality risks of all-cause and CVD.

### Supplementary Information

**Additional file 1:** **Supplementary Figure 1.** Scatter plot for the relationship between daily eating frequency and nighttime fasting duration. **Supplementary Figure 2.** Dose-response association for the Daily eating frequency and nighttime fasting duration and mortalities for heart-disease specific and stroke specific. **Supplementary Table 1.** Association of daily eating frequency with mortalities of all-cause, CVD, heart disease and stroke after excluding the participants whose follow-up years were less than two years. **Supplementary Table 2.** Association of nighttime fasting duration with mortalities of all-cause, CVD, heart disease and stroke after excluding the participants whose follow-up years were less than two years. **Supplementary Table 3.** Association of daily eating frequency with mortalities of all-cause, CVD, heart disease and stroke after excluding the participants who did not eat breakfast. **Supplementary Table 4.** Association of nighttime fasting duration with mortalities of all-cause, CVD, heart disease and stroke after excluding the participants who did not eat breakfast. **Supplementary Table 5.** Association of daily eating frequency with mortalities of all-cause, CVD, heart disease and stroke after excluding the participants who did not eat lunch. **Supplementary Table 6.** Association of nighttime fasting duration with mortalities of all-cause, CVD, heart disease and stroke after excluding the participants who did not eat lunch. **Supplementary Table 7.** Association of daily eating frequency with mortalities of all-cause, CVD, heart disease and stroke after excluding the participants who did not eat dinner. **Supplementary Table 8.** Association of nighttime fasting duration with mortalities of all-cause, CVD, heart disease and stroke after excluding the participants who did not eat dinner. **Supplementary Table 9.** The p-values for the modification effects of potential confounders on the association of daily eating frequency and nighttime fasting duration with mortality outcomes. **Supplementary Table 10.** Association of daily eating frequency with mortalities of all-cause and CVD among participants with AHEI below or over median.

## Data Availability

The National Health and Nutrition Examination Survey (NHANES) data are publicly available at https://www.cdc.gov/nchs/nhanes/index.htm.

## Abbreviations

DEF: Daily eating frequency
NFD: Nighttime fasting duration
CVD: Cardiovascular disease
HR: Hazard-ratio
Cl: Confidence-interval
AHEI: Alternative Healthy eating index
TG: Plasma triglycerides
TC: Plasma total cholesterol
CPH: Cox proportional hazards
