# Supplementary material for: Relationship between circadian eating behavior (daily eating frequency and nighttime fasting duration) and cardiovascular mortality
Source: Int J Behav Nutr Phys Act. 2024 Feb 26;21:22. doi: 10.1186/s12966-023-01556-5 (PMC10895826; doi:10.1186/s12966-023-01556-5)
Supplement: Supplementary file 1 — Additional file 1: Supplementary Figure 1. Scatter plot for the relationship between daily eating frequency and nighttime fasting duration. Supplementary Figure 2. Dose-response association for the Daily eating frequency and nighttime fasting duration and mortalities for heart-disease specific and stroke specific. Supplementary Table 1. Association of daily eating frequency with mortalities of all-cause, CVD, heart disease and stroke after excluding the participants whose follow-up years were less than two years. Supplementary Table 2. Association of nighttime fasting duration with mortalities of all-cause, CVD, heart disease and stroke after excluding the participants whose follow-up years were less than two years. Supplementary Table 3. Association of daily eating frequency with mortalities of all-cause, CVD, heart disease and stroke after excluding the participants who did not eat breakfast. Supplementary Table 4. Association of nighttime fasting duration with mortalities of all-cause, CVD, heart disease and stroke after excluding the participants who did not eat breakfast. Supplementary Table 5. Association of daily eating frequency with mortalities of all-cause, CVD, heart disease and stroke after excluding the participants who did not eat lunch. Supplementary Table 6. Association of nighttime fasting duration with mortalities of all-cause, CVD, heart disease and stroke after excluding the participants who did not eat lunch. Supplementary Table 7. Association of daily eating frequency with mortalities of all-cause, CVD, heart disease and stroke after excluding the participants who did not eat dinner. Supplementary Table 8. Association of nighttime fasting duration with mortalities of all-cause, CVD, heart disease and stroke after excluding the participants who did not eat dinner. Supplementary Table 9. The p-values for the modification effects of potential confounders on the association of daily eating frequency and nighttime fasting duration with mortality out [file 12966_2023_1556_MOESM1_ESM.docx]

Supplementary Figure 1- Scatter plot for the relationship between daily eating frequency and nighttime fasting duration


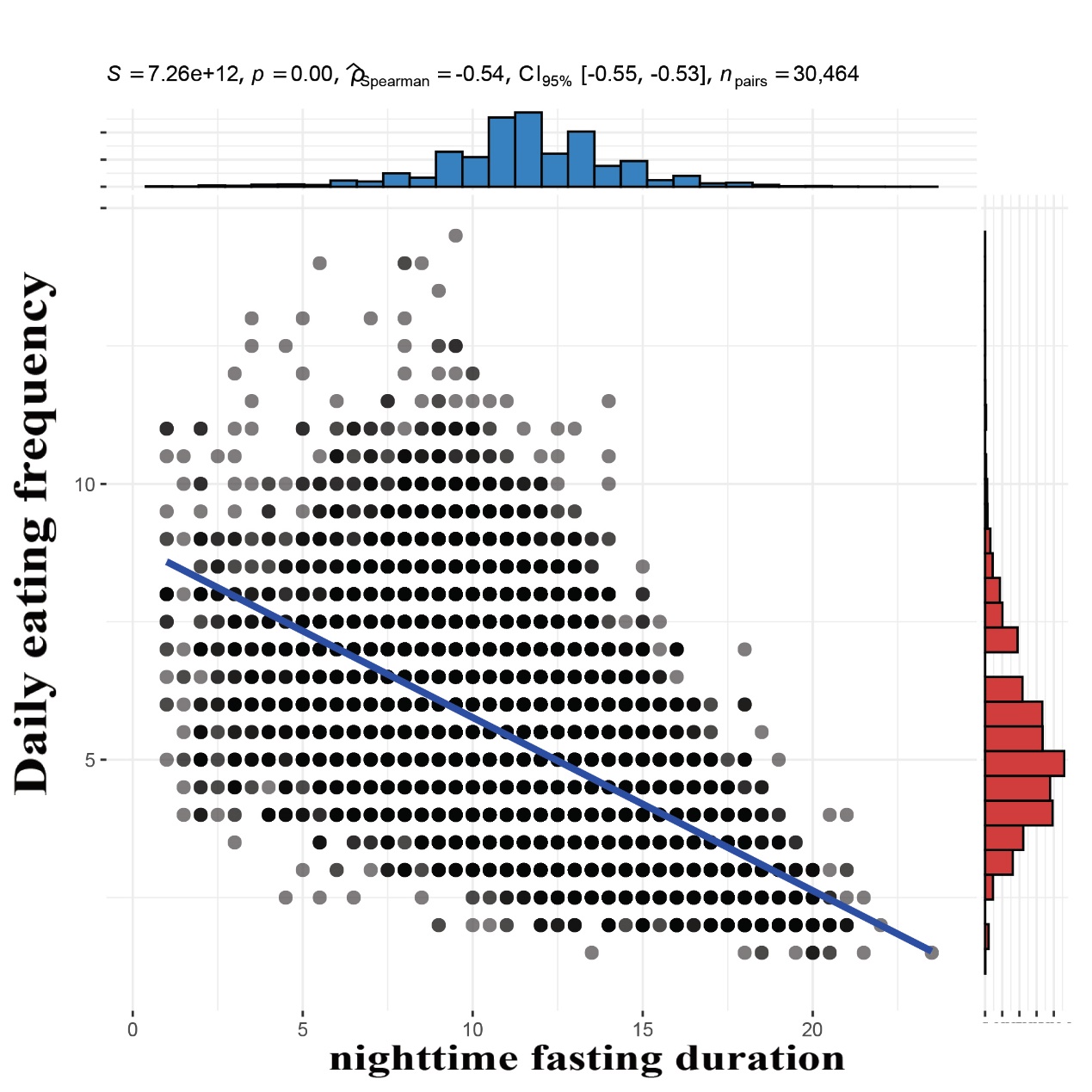


Supplementary Figure 2- Dose-response association for the Daily eating frequency and nighttime fasting duration and mortalities for heart-disease specific and stroke specific


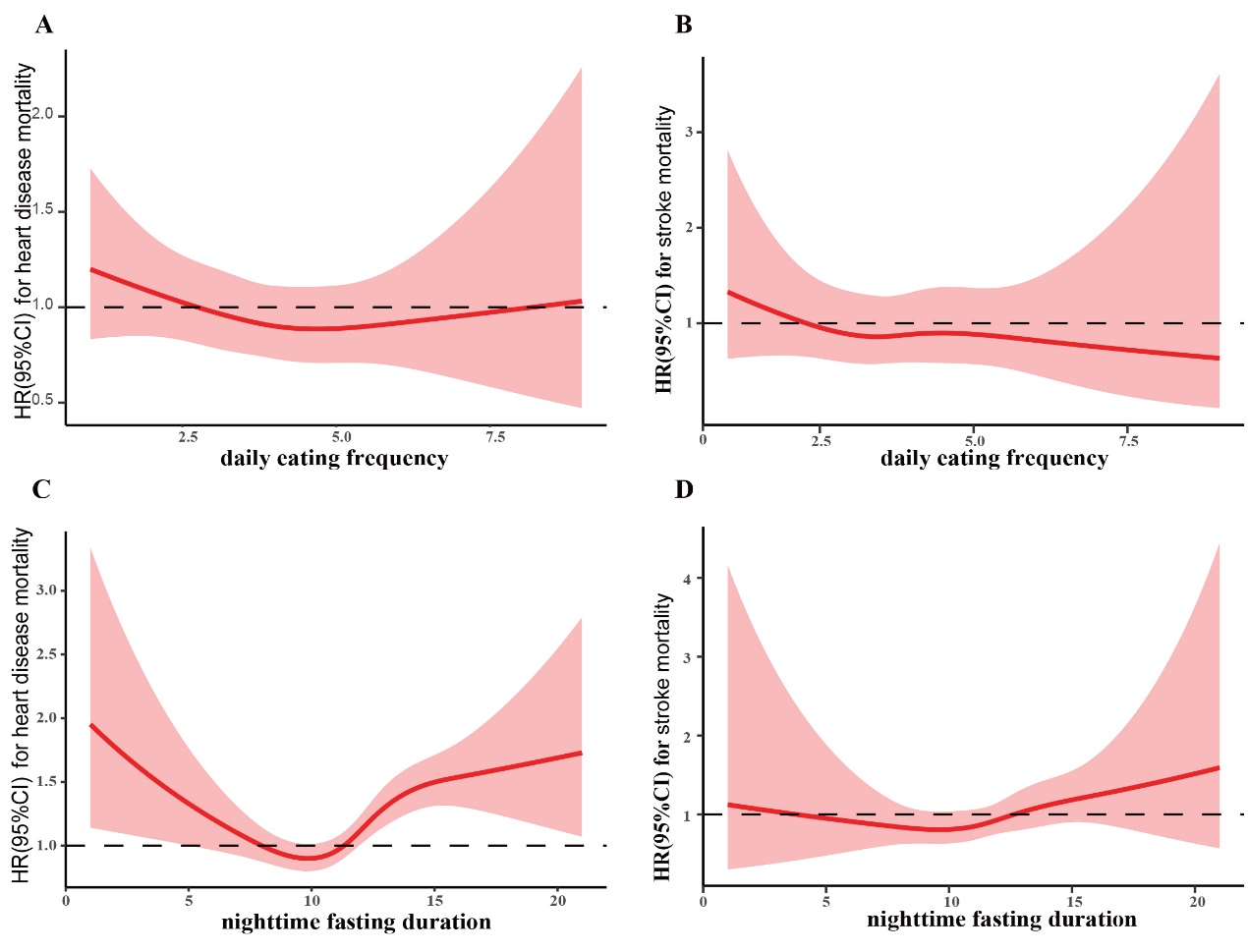


Figure legends: (A) daily eating frequency and heart-disease specific mortality; (B) daily eating frequency and stroke specific mortality; (C) nighttime fasting duration and heart-disease specific mortality; (D) nighttime fasting duration and stroke-specific mortality

Supplementary Table 1-Association of daily eating frequency with mortalities of all-cause, CVD, heart disease and stroke after excluding the participants whose follow-up years were less than two years

|  | Daily eating Frequency | | | | |
| --- | --- | --- | --- | --- | --- |
|  | Quintile 1  (< 3 times) | Quintile 2  (3 to 3.5 times) | Quintile 3  (3.5 to 4 times) | Quintile 4  (4 to 4.5 times) | Quintile 5  (4.5 to 6 times) |
| All-cause mortality |  |  |  |  |  |
| Death/person-years | 1626/101510 | 668/47590 | 684/53740 | 446/35590 | 558/58132 |
| Unadjusted | 1.67 (1.48-1.89) | 1.54 (1.34-1.77) | 1.39 (1.21-1.59) | 1.44 (1.25-1.66) | 1 (ref.) |
| Model 1 | 1.38 (1.21-1.56) | 1.30 (1.13-1.49) | 1.22 (1.06-1.42) | 1.23 (1.06-1.42) | 1 (ref.) |
| Model 2 | 1.22 (1.07-1.40) | 1.23 (1.07-1.41) | 1.16 (0.99-1.34) | 1.21 (1.04-1.39) | 1 (ref.) |
| Model 3 | 1.16 (1.01-1.33) | 1.19 (1.05-1.37) | 1.14 (0.98-1.33) | 1.21 (1.04-1.39) | 1 (ref.) |
| CVD mortality |  |  |  |  |  |
| Death/person-years | 694/101510 | 269/47590 | 274/53740 | 165/35590 | 199/58132 |
| Unadjusted | 2.12 (1.75-2.57) | 1.79 (1.44-2.21) | 1.72 (1.40-2.10) | 1.49 (1.18-1.88) | 1 (ref.) |
| Model 1 | 1.63 (1.33-2.00) | 1.41 (1.14-1.75) | 1.44 (1.17-1.77) | 1.22 (0.95-1.56) | 1 (ref.) |
| Model 2 | 1.39 (1.12-1.71) | 1.28 (1.04-1.59) | 1.33 (1.08-1.63) | 1.18 (0.92-1.50) | 1 (ref.) |
| Model 3 | 1.33 (1.06-1.67) | 1.27 (1.02-1.58) | 1.32 (1.08-1.63) | 1.18 (0.92-1.51) | 1 (ref.) |
| Heart disease-specific mortality |  |  |  |  |  |
| Death/person-years | 436/101510 | 172/47590 | 188/53740 | 102/35590 | 129/58132 |
| Unadjusted | 2.35 (1.86-2.98) | 2.06 (1.57-2.69) | 2.07 (1.62-2.64) | 1.64 (1.24-2.18) | 1 (ref.) |
| Model 1 | 1.87 (1.47-2.39) | 1.64 (1.24-2.15) | 1.80 (1.41-2.30) | 1.36 (1.03-1.81) | 1 (ref.) |
| Model 2 | 1.51 (1.17-1.94) | 1.44 (1.10-1.89) | 1.62 (1.27-2.07) | 1.29 (0.98-1.71) | 1 (ref.) |
| Model 3 | 1.44 (1.08-1.90) | 1.43 (1.07-1.91) | 1.62 (1.25-2.09) | 1.30 (0.98-1.74) | 1 (ref.) |
| Stroke-specific mortality |  |  |  |  |  |
| Death/person-years | 102/101510 | 29/47590 | 41/53740 | 26/35590 | 28/58132 |
| Unadjusted | 2.30 (1.42-3.70) | 1.51 (0.85-2.68) | 1.62 (0.98-2.70) | 1.72 (0.95-3.11) | 1 (ref.) |
| Model 1 | 1.73 (1.09-2.76) | 1.21 (0.67-2.15) | 1.22 (0.73-2.04) | 1.42 (0.78-2.58) | 1 (ref.) |
| Model 2 | 1.54 (0.94-2.51) | 1.12 (0.64-1.96) | 1.16 (0.69-1.95) | 1.38 (0.77-2.46) | 1 (ref.) |
| Model 3 | 1.56 (0.90-2.71) | 1.15 (0.67-1.97) | 1.19 (0.71-2.00) | 1.42 (0.80-2.53) | 1 (ref.) |

Data is weighted HR and 95%CI.

Model 1 is unadjusted model with additionally adjusted for age, age, sex, race, education level, annual family income, regular exercise habitus, smoking, drinking, BMI, sleep duration, daily energy intake, AHEI, meal skipping and whether dietary data was surveyed on weekend.

Model 2 is model 1 with additionally adjusted for fasting plasma glucose, TG, TC, drug use for controlling glucose/ hypertension/ dyslipidemia, family history of CVD.

Model 3 is model 2 with additionally adjusted for nighttime fasting duration.

Supplementary Table 2-Association of nighttime fasting duration with mortalities of all-cause, CVD, heart disease and stroke after excluding the participants whose follow-up years were less than two years

|  | Nighttime fasting duration | | | | |
| --- | --- | --- | --- | --- | --- |
|  | Quintile 1  (< 10 hours) | Quintile 2  (10 to 11 hours) | Quintile 3  (11 to 12 hours) | Quintile 4  (12 to 14 hours) | Quintile 5  (> 14 hours) |
| All-cause mortality |  |  |  |  |  |
| Death/person-years | 901/77760 | 607/51005 | 721/55009 | 917/65467 | 838/55789 |
| Unadjusted | 1.05(0.91-1.21) | 1 (ref.) | 1.17(1.01-1.36) | 1.20(1.04-1.38) | 1.30(1.13-1.49) |
| Model 1 | 1.16(1.00-1.33) | 1 (ref.) | 1.18(1.02-1.35) | 1.25(1.11-1.42) | 1.54(1.35-1.77) |
| Model 2 | 1.18(1.03-1.36) | 1 (ref.) | 1.14(0.99-1.32) | 1.20(1.06-1.36) | 1.44(1.26-1.66) |
| Model 3 | 1.19(1.03-1.35) | 1 (ref.) | 1.15(1.00-1.33) | 1.20(1.05-1.36) | 1.45(1.26-1.67) |
| CVD mortality |  |  |  |  |  |
| Death/person-years | 344/77760 | 240/51005 | 285/55009 | 371/65467 | 361/55789 |
| Unadjusted | 1.04(0.85-1.26) | 1 (ref.) | 1.20(0.98-1.48) | 1.20(0.96-1.49) | 1.42(1.17-1.71) |
| Model 1 | 1.17(0.97-1.40) | 1 (ref.) | 1.21(0.99-1.48) | 1.23(1.00-1.53) | 1.62(1.33-1.97) |
| Model 2 | 1.20(1.00-1.44) | 1 (ref.) | 1.16(0.95-1.42) | 1.15(0.93-1.42) | 1.43(1.19-1.73) |
| Model 3 | 1.20(1.00-1.44) | 1 (ref.) | 1.18(0.97-1.43) | 1.15(0.93-1.42) | 1.46(1.20-1.76) |
| Heart disease-specific mortality |  |  |  |  |  |
| Death/person-years | 225/77760 | 155/51005 | 181/55009 | 233/65467 | 233/55789 |
| Unadjusted | 1.09(0.84-1.40) | 1 (ref.) | 1.25(0.96-1.63) | 1.21(0.94-1.55) | 1.53(1.21-1.95) |
| Model 1 | 1.21(0.95-1.55) | 1 (ref.) | 1.27(0.98-1.64) | 1.26(0.99-1.60) | 1.81(1.39-2.36) |
| Model 2 | 1.26(0.98-1.62) | 1 (ref.) | 1.20(0.93-1.56) | 1.15(0.90-1.45) | 1.55(1.20-2.02) |
| Model 3 | 1.22(0.94-1.58) | 1 (ref.) | 1.15(0.89-1.50) | 1.13(0.88-1.44) | 1.50(1.15-1.95) |
| Stroke-specific mortality |  |  |  |  |  |
| Death/person-years | 50/77760 | 28/51005 | 39/55009 | 59/65467 | 50/55789 |
| Unadjusted | 1.54(0.95-2.50) | 1 (ref.) | 1.51(0.87-2.62) | 1.87(1.08-3.25) | 1.85(1.12-3.05) |
| Model 1 | 1.76(1.07-2.89) | 1 (ref.) | 1.46(0.83-2.55) | 1.87(1.06-3.29) | 2.08(1.26-3.44) |
| Model 2 | 1.82(1.11-3.02) | 1 (ref.) | 1.39(0.80-2.44) | 1.73(0.96-3.10) | 1.83(1.10-3.04) |
| Model 3 | 1.82(1.10-2.99) | 1 (ref.) | 1.41(0.81-2.47) | 1.74(0.96-3.14) | 1.86(1.12-3.08) |

Data is weighted HR and 95%CI.

Model 1 is unadjusted model with additionally adjusted for age, age, sex, race, education level, annual family income, regular exercise habitus, smoking, drinking, BMI, sleep duration, daily energy intake, AHEI, meal skipping and whether dietary data was surveyed on weekend.

Model 2 is model 1 with additionally adjusted for fasting plasma glucose, TG, TC, drug use for controlling glucose/ hypertension/ dyslipidemia, family history of CVD.

Model 3 is model 2 with additionally adjusted for daily eating frequency.

Supplementary Table 3-Association of daily eating frequency with mortalities of all-cause, CVD, heart disease and stroke after excluding the participants who did not eat breakfast

|  | Daily eating Frequency | | | | |
| --- | --- | --- | --- | --- | --- |
|  | Quintile 1  (< 3 times) | Quintile 2  (3 to 3.5 times) | Quintile 3  (3.5 to 4 times) | Quintile 4  (4 to 4.5 times) | Quintile 5  (4.5 to 6 times) |
| All-cause mortality |  |  |  |  |  |
| Death/person-years | 1817/97833 | 743/47005 | 776/51787 | 490/34917 | 627/56486 |
| Unadjusted | 1.75 (1.56-1.98) | 1.53 (1.33-1.75) | 1.40 (1.22-1.60) | 1.42 (1.23-1.65) | 1 (ref.) |
| Model 1 | 1.37 (1.21-1.56) | 1.28 (1.12-1.47) | 1.22 (1.05-1.41) | 1.21 (1.05-1.40) | 1 (ref.) |
| Model 2 | 1.22 (1.07-1.39) | 1.21 (1.05-1.39) | 1.15 (0.99-1.33) | 1.19 (1.03-1.38) | 1 (ref.) |
| Model 3 | 1.16 (1.01-1.33) | 1.18 (1.03-1.36) | 1.14 (0.98-1.32) | 1.19 (1.03-1.38) | 1 (ref.) |
| CVD mortality |  |  |  |  |  |
| Death/person-years | 774/97833 | 288/47005 | 311/51787 | 180/34917 | 226/56486 |
| Unadjusted | 2.22 (1.84-2.69) | 1.73 (1.41-2.14) | 1.70 (1.39-2.08) | 1.47 (1.16-1.87) | 1 (ref.) |
| Model 1 | 1.63 (1.33-2.00) | 1.36 (1.10-1.69) | 1.41 (1.16-1.73) | 1.20 (0.93-1.53) | 1 (ref.) |
| Model 2 | 1.38 (1.12-1.70) | 1.24 (1.00-1.53) | 1.30 (1.06-1.60) | 1.16 (0.91-1.48) | 1 (ref.) |
| Model 3 | 1.34 (1.07-1.67) | 1.23 (0.98-1.53) | 1.30 (1.06-1.60) | 1.16 (0.91-1.49) | 1 (ref.) |
| Heart disease-specific mortality |  |  |  |  |  |
| Death/person-years | 496/97833 | 190/47005 | 213/51787 | 113/34917 | 142/56486 |
| Unadjusted | 2.44 (1.93-3.09) | 1.99 (1.53-2.58) | 2.06 (1.61-2.64) | 1.63 (1.23-2.16) | 1 (ref.) |
| Model 1 | 1.85 (1.45-2.38) | 1.58 (1.21-2.06) | 1.77 (1.38-2.27) | 1.35 (1.02-1.79) | 1 (ref.) |
| Model 2 | 1.49 (1.16-1.91) | 1.38 (1.06-1.81) | 1.60 (1.24-2.04) | 1.28 (0.97-1.69) | 1 (ref.) |
| Model 3 | 1.43 (1.07-1.90) | 1.38 (1.04-1.84) | 1.60 (1.23-2.07) | 1.29 (0.97-1.72) | 1 (ref.) |
| Stroke-specific mortality |  |  |  |  |  |
| Death/person-years | 110/97833 | 30/47005 | 44/51787 | 28/34917 | 33/56486 |
| Unadjusted | 2.41 (1.50-3.85) | 1.41 (0.78-2.55) | 1.53 (0.92-2.54) | 1.71 (0.94-3.11) | 1 (ref.) |
| Model 1 | 1.75 (1.11-2.76) | 1.10 (0.61-2.00) | 1.11 (0.67-1.83) | 1.40 (0.77-2.54) | 1 (ref.) |
| Model 2 | 1.57 (0.97-2.55) | 1.04 (0.58-1.85) | 1.05 (0.63-1.76) | 1.37 (0.77-2.46) | 1 (ref.) |
| Model 3 | 1.62 (0.94-2.79) | 1.08 (0.62-1.88) | 1.09 (0.65-1.83) | 1.44 (0.81-2.55) | 1 (ref.) |

Data is weighted HR and 95%CI.

Model 1 is unadjusted model with additionally adjusted for age, age, sex, race, education level, annual family income, regular exercise habitus, smoking, drinking, BMI, sleep duration, daily energy intake, AHEI, meal skipping and whether dietary data was surveyed on weekend.

Model 2 is model 1 with additionally adjusted for fasting plasma glucose, TG, TC, drug use for controlling glucose/ hypertension/ dyslipidemia, family history of CVD.

Model 3 is model 2 with additionally adjusted for nighttime fasting duration.

Supplementary Table 4-Association of nighttime fasting duration with mortalities of all-cause, CVD, heart disease and stroke after excluding the participants who did not eat breakfast

|  | Nighttime fasting duration | | | | |
| --- | --- | --- | --- | --- | --- |
|  | Quintile 1  (< 10 hours) | Quintile 2  (10 to 11 hours) | Quintile 3  (11 to 12 hours) | Quintile 4  (12 to 14 hours) | Quintile 5  (> 14 hours) |
| All-cause mortality |  |  |  |  |  |
| Death/person-years | 1005/75660 | 671/49480 | 802/53500 | 1049/62852 | 929/46611 |
| Unadjusted | 1.08(0.95-1.23) | 1 (ref.) | 1.17(1.02-1.34) | 1.25(1.09-1.43) | 1.51(1.32-1.72) |
| Model 1 | 1.18(1.04-1.34) | 1 (ref.) | 1.18(1.04-1.34) | 1.29(1.15-1.46) | 1.63(1.43-1.85) |
| Model 2 | 1.20(1.06-1.36) | 1 (ref.) | 1.15(1.01-1.31) | 1.24(1.10-1.39) | 1.50(1.31-1.71) |
| Model 3 | 1.17(1.03-1.33) | 1 (ref.) | 1.12(0.98-1.28) | 1.22(1.08-1.37) | 1.44(1.26-1.65) |
| CVD mortality |  |  |  |  |  |
| Death/person-years | 381/75660 | 258/49480 | 320/53500 | 427/62852 | 394/46611 |
| Unadjusted | 1.11(0.92-1.34) | 1 (ref.) | 1.24(1.01-1.53) | 1.32(1.07-1.62) | 1.68(1.39-2.02) |
| Model 1 | 1.24(1.04-1.48) | 1 (ref.) | 1.25(1.03-1.52) | 1.34(1.09-1.64) | 1.72(1.43-2.06) |
| Model 2 | 1.28(1.08-1.52) | 1 (ref.) | 1.20(0.99-1.45) | 1.24(1.01-1.53) | 1.52(1.26-1.82) |
| Model 3 | 1.22(1.02-1.47) | 1 (ref.) | 1.18(0.97-1.43) | 1.23(0.99-1.52) | 1.51(1.26-1.81) |
| Heart disease-specific mortality |  |  |  |  |  |
| Death/person-years | 252/75660 | 165/49480 | 204/53500 | 272/62852 | 262/46611 |
| Unadjusted | 1.20(0.94-1.53) | 1 (ref.) | 1.31(1.00-1.70) | 1.35(1.07-1.70) | 1.90(1.50-2.40) |
| Model 1 | 1.33(1.06-1.68) | 1 (ref.) | 1.33(1.03-1.72) | 1.39(1.10-1.75) | 2.01(1.57-2.60) |
| Model 2 | 1.39(1.10-1.75) | 1 (ref.) | 1.26(0.97-1.63) | 1.26(1.01-1.58) | 1.71(1.34-2.20) |
| Model 3 | 1.38(1.10-1.74) | 1 (ref.) | 1.27(0.99-1.64) | 1.26(1.00-1.57) | 1.73(1.35-2.22) |
| Stroke-specific mortality |  |  |  |  |  |
| Death/person-years | 57/75660 | 30/49480 | 42/53500 | 65/62852 | 51/46611 |
| Unadjusted | 1.66(1.03-2.66) | 1 (ref.) | 1.48(0.85-2.56) | 1.92(1.12-3.27) | 1.97(1.22-3.15) |
| Model 1 | 1.88(1.16-3.05) | 1 (ref.) | 1.42(0.82-2.47) | 1.87(1.09-3.22) | 1.95(1.22-3.11) |
| Model 2 | 1.92(1.19-3.09) | 1 (ref.) | 1.38(0.79-2.42) | 1.77(0.99-3.17) | 1.79(1.10-2.92) |
| Model 3 | 1.38(1.10-1.74) | 1 (ref.) | 1.27(0.99-1.64) | 1.26(1.00-1.57) | 1.73(1.35-2.22) |

Data is weighted HR and 95%CI.

Model 1 is unadjusted model with additionally adjusted for age, age, sex, race, education level, annual family income, regular exercise habitus, smoking, drinking, BMI, sleep duration, daily energy intake, AHEI, meal skipping and whether dietary data was surveyed on weekend.

Model 2 is model 1 with additionally adjusted for fasting plasma glucose, TG, TC, drug use for controlling glucose/ hypertension/ dyslipidemia, family history of CVD.

Model 3 is model 2 with additionally adjusted for daily eating frequency.

Supplementary Table 5-Association of daily eating frequency with mortalities of all-cause, CVD, heart disease and stroke after excluding the participants who did not eat lunch

|  | Daily eating Frequency | | | | |
| --- | --- | --- | --- | --- | --- |
|  | Quintile 1  (< 3 times) | Quintile 2  (3 to 3.5 times) | Quintile 3  (3.5 to 4 times) | Quintile 4  (4 to 4.5 times) | Quintile 5  (4.5 to 6 times) |
| All-cause mortality |  |  |  |  |  |
| Death/person-years | 1499/91864 | 678/45319 | 697/48728 | 460/33472 | 581/54320 |
| Unadjusted | 1.61 (1.41-1.85) | 1.52 (1.32-1.76) | 1.40 (1.21-1.61) | 1.45 (1.24-1.68) | 1 (ref.) |
| Model 1 | 1.35 (1.18-1.55) | 1.31 (1.13-1.52) | 1.24 (1.06-1.44) | 1.25 (1.07-1.46) | 1 (ref.) |
| Model 2 | 1.21 (1.04-1.40) | 1.22 (1.05-1.42) | 1.17 (1.00-1.37) | 1.22 (1.04-1.43) | 1 (ref.) |
| Model 3 | 1.14 (0.98-1.33) | 1.19 (1.02-1.38) | 1.15 (0.97-1.35) | 1.21 (1.03-1.42) | 1 (ref.) |
| CVD mortality |  |  |  |  |  |
| Death/person-years | 640/91864 | 264/45319 | 285/48728 | 165/33472 | 203/54320 |
| Unadjusted | 2.10 (1.69-2.61) | 1.80 (1.43-2.26) | 1.78 (1.45-2.18) | 1.49 (1.16-1.91) | 1 (ref.) |
| Model 1 | 1.64 (1.31-2.04) | 1.44 (1.14-1.81) | 1.49 (1.20-1.84) | 1.23 (0.95-1.60) | 1 (ref.) |
| Model 2 | 1.38 (1.09-1.75) | 1.28 (1.01-1.62) | 1.37 (1.10-1.71) | 1.17 (0.90-1.52) | 1 (ref.) |
| Model 3 | 1.34 (1.04-1.73) | 1.27 (1.00-1.62) | 1.37 (1.09-1.71) | 1.17 (0.90-1.53) | 1 (ref.) |
| Heart disease-specific mortality |  |  |  |  |  |
| Death/person-years | 405/91864 | 170/45319 | 198/48728 | 104/33472 | 126/54320 |
| Unadjusted | 2.35 (1.79-3.07) | 2.09 (1.57-2.77) | 2.20 (1.70-2.84) | 1.72 (1.26-2.35) | 1 (ref.) |
| Model 1 | 1.88 (1.43-2.48) | 1.66 (1.24-2.24) | 1.90 (1.46-2.47) | 1.44 (1.06-1.98) | 1 (ref.) |
| Model 2 | 1.53 (1.14-2.04) | 1.45 (1.08-1.94) | 1.72 (1.32-2.25) | 1.36 (0.99-1.85) | 1 (ref.) |
| Model 3 | 1.47 (1.06-2.04) | 1.45 (1.06-1.97) | 1.72 (1.31-2.27) | 1.36 (0.99-1.88) | 1 (ref.) |
| Stroke-specific mortality |  |  |  |  |  |
| Death/person-years | 96/91864 | 30/45319 | 38/48728 | 26/33472 | 28/54320 |
| Unadjusted | 2.48 (1.42-4.32) | 1.76 (0.95-3.26) | 1.66 (0.96-2.88) | 1.88 (1.00-3.54) | 1 (ref.) |
| Model 1 | 1.92 (1.13-3.27) | 1.44 (0.77-2.70) | 1.34 (0.76-2.37) | 1.58 (0.83-2.98) | 1 (ref.) |
| Model 2 | 1.71 (0.96-3.03) | 1.33 (0.72-2.46) | 1.27 (0.71-2.26) | 1.53 (0.82-2.87) | 1 (ref.) |
| Model 3 | 1.74 (0.94-3.25) | 1.37 (0.76-2.46) | 1.31 (0.73-2.34) | 1.58 (0.85-2.96) | 1 (ref.) |

Data is weighted HR and 95%CI.

Model 1 is unadjusted model with additionally adjusted for age, age, sex, race, education level, annual family income, regular exercise habitus, smoking, drinking, BMI, sleep duration, daily energy intake, AHEI, meal skipping and whether dietary data was surveyed on weekend.

Model 2 is model 1 with additionally adjusted for fasting plasma glucose, TG, TC, drug use for controlling glucose/ hypertension/ dyslipidemia, family history of CVD.

Model 3 is model 2 with additionally adjusted for nighttime fasting duration.

Supplementary Table 6-Association of nighttime fasting duration with mortalities of all-cause, CVD, heart disease and stroke after excluding the participants who did not eat lunch

|  | Nighttime fasting duration | | | | |
| --- | --- | --- | --- | --- | --- |
|  | Quintile 1  (< 10 hours) | Quintile 2  (10 to 11 hours) | Quintile 3  (11 to 12 hours) | Quintile 4  (12 to 14 hours) | Quintile 5  (> 14 hours) |
| All-cause mortality |  |  |  |  |  |
| Death/person-years | 870/70460 | 601/47329 | 713/49439 | 938/58711 | 795/48111 |
| Unadjusted | 1.05(0.92-1.20) | 1 (ref.) | 1.19(1.03-1.39) | 1.23(1.06-1.42) | 1.34(1.18-1.52) |
| Model 1 | 1.16(1.02-1.32) | 1 (ref.) | 1.20(1.05-1.38) | 1.30(1.15-1.47) | 1.59(1.39-1.81) |
| Model 2 | 1.19(1.05-1.35) | 1 (ref.) | 1.17(1.02-1.35) | 1.25(1.10-1.41) | 1.50(1.31-1.72) |
| Model 3 | 1.15(1.01-1.30) | 1 (ref.) | 1.15(0.99-1.33) | 1.23(1.08-1.40) | 1.44(1.26-1.65) |
| CVD mortality |  |  |  |  |  |
| Death/person-years | 332/70460 | 228/47329 | 290/49439 | 377/58711 | 331/48111 |
| Unadjusted | 1.08(0.89-1.31) | 1 (ref.) | 1.32(1.06-1.64) | 1.28(1.04-1.58) | 1.48(1.23-1.79) |
| Model 1 | 1.22(1.02-1.45) | 1 (ref.) | 1.34(1.09-1.64) | 1.33(1.08-1.64) | 1.71(1.41-2.08) |
| Model 2 | 1.27(1.07-1.51) | 1 (ref.) | 1.29(1.05-1.58) | 1.23(1.00-1.52) | 1.50(1.25-1.80) |
| Model 3 | 1.19(0.99-1.43) | 1 (ref.) | 1.23(1.00-1.51) | 1.21(0.97-1.49) | 1.44(1.19-1.73) |
| Heart disease-specific mortality |  |  |  |  |  |
| Death/person-years | 218/70460 | 145/47329 | 183/49439 | 235/58711 | 223/48111 |
| Unadjusted | 1.17(0.91-1.51) | 1 (ref.) | 1.39(1.06-1.83) | 1.29(1.02-1.65) | 1.70(1.35-2.16) |
| Model 1 | 1.32(1.03-1.67) | 1 (ref.) | 1.42(1.08-1.85) | 1.36(1.07-1.72) | 2.01(1.55-2.61) |
| Model 2 | 1.39(1.09-1.77) | 1 (ref.) | 1.35(1.04-1.76) | 1.24(0.98-1.56) | 1.73(1.35-2.23) |
| Model 3 | 1.38(1.09-1.75) | 1 (ref.) | 1.35(1.04-1.76) | 1.23(0.98-1.54) | 1.75(1.36-2.26) |
| Stroke-specific mortality |  |  |  |  |  |
| Death/person-years | 50/70460 | 27/47329 | 37/49439 | 60/58711 | 44/48111 |
| Unadjusted | 1.61(0.97-2.68) | 1 (ref.) | 1.51(0.80-2.84) | 1.90(1.09-3.30) | 1.80(1.09-2.97) |
| Model 1 | 1.84(1.09-3.10) | 1 (ref.) | 1.49(0.79-2.78) | 1.96(1.11-3.44) | 2.07(1.26-3.39) |
| Model 2 | 1.87(1.11-3.16) | 1 (ref.) | 1.44(0.76-2.72) | 1.82(1.01-3.30) | 1.79(1.06-3.01) |
| Model 3 | 1.38(1.09-1.75) | 1 (ref.) | 1.35(1.04-1.76) | 1.23(0.98-1.54) | 1.75(1.36-2.26) |

Data is weighted HR and 95%CI.

Model 1 is unadjusted model with additionally adjusted for age, age, sex, race, education level, annual family income, regular exercise habitus, smoking, drinking, BMI, sleep duration, daily energy intake, AHEI, meal skipping and whether dietary data was surveyed on weekend.

Model 2 is model 1 with additionally adjusted for fasting plasma glucose, TG, TC, drug use for controlling glucose/ hypertension/ dyslipidemia, family history of CVD.

Model 3 is model 2 with additionally adjusted for daily eating frequency.

Supplementary Table 7-Association of daily eating frequency with mortalities of all-cause, CVD, heart disease and stroke after excluding the participants who did not eat dinner

|  | Daily eating Frequency | | | | |
| --- | --- | --- | --- | --- | --- |
|  | Quintile 1  (< 3 times) | Quintile 2  (3 to 3.5 times) | Quintile 3  (3.5 to 4 times) | Quintile 4  (4 to 4.5 times) | Quintile 5  (4.5 to 6 times) |
| All-cause mortality |  |  |  |  |  |
| Death/person-years | 1806/105116 | 744/48015 | 771/52906 | 480/35270 | 617/56966 |
| Unadjusted | 1.67 (1.48-1.88) | 1.55 (1.35-1.78) | 1.40 (1.22-1.60) | 1.42 (1.23-1.64) | 1 (ref.) |
| Model 1 | 1.38 (1.22-1.57) | 1.32 (1.15-1.51) | 1.23 (1.06-1.43) | 1.22 (1.05-1.41) | 1 (ref.) |
| Model 2 | 1.23 (1.08-1.41) | 1.24 (1.08-1.42) | 1.16 (1.00-1.35) | 1.19 (1.03-1.38) | 1 (ref.) |
| Model 3 | 1.17 (1.02-1.34) | 1.21 (1.06-1.39) | 1.15 (0.99-1.34) | 1.19 (1.03-1.38) | 1 (ref.) |
| CVD mortality |  |  |  |  |  |
| Death/person-years | 765/105116 | 291/48015 | 310/52906 | 175/35270 | 218/56966 |
| Unadjusted | 2.15 (1.76-2.62) | 1.83 (1.47-2.27) | 1.74 (1.41-2.15) | 1.48 (1.16-1.89) | 1 (ref.) |
| Model 1 | 1.67 (1.36-2.06) | 1.45 (1.17-1.81) | 1.47 (1.19-1.81) | 1.22 (0.94-1.58) | 1 (ref.) |
| Model 2 | 1.42 (1.15-1.76) | 1.32 (1.06-1.64) | 1.35 (1.10-1.67) | 1.17 (0.91-1.51) | 1 (ref.) |
| Model 3 | 1.37 (1.09-1.73) | 1.30 (1.04-1.64) | 1.35 (1.09-1.67) | 1.17 (0.90-1.52) | 1 (ref.) |
| Heart disease-specific mortality |  |  |  |  |  |
| Death/person-years | 490/105116 | 189/48015 | 213/52906 | 108/35270 | 136/56966 |
| Unadjusted | 2.38 (1.86-3.04) | 2.09 (1.59-2.73) | 2.08 (1.62-2.67) | 1.60 (1.19-2.15) | 1 (ref.) |
| Model 1 | 1.91 (1.49-2.46) | 1.67 (1.27-2.20) | 1.83 (1.43-2.33) | 1.33(0.99-1.79) | 1 (ref.) |
| Model 2 | 1.56 (1.21-2.00) | 1.47 (1.12-1.94) | 1.65 (1.29-2.11) | 1.26 (0.94-1.69) | 1 (ref.) |
| Model 3 | 1.50 (1.13-1.98) | 1.47 (1.10-1.96) | 1.66 (1.28-2.14) | 1.27 (0.94-1.71) | 1 (ref.) |
| Stroke-specific mortality |  |  |  |  |  |
| Death/person-years | 108/105116 | 31/48015 | 44/52906 | 28/35270 | 31/56966 |
| Unadjusted | 2.34 (1.46-3.76) | 1.59 (0.89-2.85) | 1.70 (1.02-2.85) | 1.81 (0.99-3.31) | 1 (ref.) |
| Model 1 | 1.79 (1.13-2.85) | 1.28 (0.71-2.31) | 1.29 (0.77-2.18) | 1.51 (0.82-2.78) | 1 (ref.) |
| Model 2 | 1.57 (0.98-2.51) | 1.15 (0.65-2.04) | 1.22 (0.72-2.05) | 1.43 (0.79-2.59) | 1 (ref.) |
| Model 3 | 1.56 (0.93-2.61) | 1.17 (0.68-2.01) | 1.24 (0.74-2.07) | 1.47 (0.82-2.64) | 1 (ref.) |

Data is weighted HR and 95%CI.

Model 1 is unadjusted model with additionally adjusted for age, age, sex, race, education level, annual family income, regular exercise habitus, smoking, drinking, BMI, sleep duration, daily energy intake, AHEI, meal skipping and whether dietary data was surveyed on weekend.

Model 2 is model 1 with additionally adjusted for fasting plasma glucose, TG, TC, drug use for controlling glucose/ hypertension/ dyslipidemia, family history of CVD.

Model 3 is model 2 with additionally adjusted for nighttime fasting duration.

Supplementary Table 8-Association of nighttime fasting duration with mortalities of all-cause, CVD, heart disease and stroke after excluding the participants who did not eat dinner

|  | Nighttime fasting duration | | | | |
| --- | --- | --- | --- | --- | --- |
|  | Quintile 1  (< 10 hours) | Quintile 2  (10 to 11 hours) | Quintile 3  (11 to 12 hours) | Quintile 4  (12 to 14 hours) | Quintile 5  (> 14 hours) |
| All-cause mortality |  |  |  |  |  |
| Death/person-years | 998/77180 | 656/50260 | 791/53540 | 1030/64084 | 945/53152 |
| Unadjusted | 1.08(0.94-1.23) | 1 (ref.) | 1.18(1.03-1.36) | 1.23(1.08-1.41) | 1.39(1.21-1.59) |
| Model 1 | 1.18(1.04-1.34) | 1 (ref.) | 1.20(1.05-1.37) | 1.30(1.16-1.47) | 1.65(1.45-1.88) |
| Model 2 | 1.20(1.06-1.37) | 1 (ref.) | 1.17(1.02-1.33) | 1.25(1.11-1.41) | 1.55(1.36-1.77) |
| Model 3 | 1.17(1.03-1.33) | 1 (ref.) | 1.13(0.99-1.31) | 1.23(1.09-1.39) | 1.48(1.30-1.69) |
| CVD mortality |  |  |  |  |  |
| Death/person-years | 378/77180 | 253/50260 | 316/53540 | 415/64084 | 398/53152 |
| Unadjusted | 1.09(0.90-1.32) | 1 (ref.) | 1.25(1.02-1.54) | 1.28(1.03-1.58) | 1.54(1.28-1.85) |
| Model 1 | 1.22(1.02-1.45) | 1 (ref.) | 1.28(1.06-1.55) | 1.33(1.08-1.63) | 1.75(1.46-2.09) |
| Model 2 | 1.26(1.06-1.49) | 1 (ref.) | 1.23(1.01-1.48) | 1.23(1.00-1.51) | 1.54(1.29-1.84) |
| Model 3 | 1.20(1.00-1.44) | 1 (ref.) | 1.20(0.99-1.46) | 1.21(0.98-1.50) | 1.50(1.26-1.80) |
| Heart disease-specific mortality |  |  |  |  |  |
| Death/person-years | 250/77180 | 160/50260 | 201/53540 | 264/64084 | 262/53152 |
| Unadjusted | 1.20(0.94-1.54) | 1 (ref.) | 1.32(1.02-1.72) | 1.33(1.04-1.69) | 1.75(1.38-2.22) |
| Model 1 | 1.34(1.06-1.70) | 1 (ref.) | 1.36(1.05-1.76) | 1.40(1.10-1.77) | 2.06(1.61-2.64) |
| Model 2 | 1.39(1.10-1.77) | 1 (ref.) | 1.29(1.00-1.67) | 1.27(1.01-1.60) | 1.77(1.39-2.26) |
| Model 3 | 1.39(1.10-1.76) | 1 (ref.) | 1.30(1.01-1.67) | 1.26(1.00-1.59) | 1.79(1.40-2.28) |
| Stroke-specific mortality |  |  |  |  |  |
| Death/person-years | 54/77180 | 29/50260 | 43/53540 | 63/64084 | 53/53152 |
| Unadjusted | 1.49(0.91-2.44) | 1 (ref.) | 1.55(0.87-2.75) | 1.78(1.04-30.6) | 1.78(1.12-2.83) |
| Model 1 | 1.69(1.03-2.78) | 1 (ref.) | 1.52(0.86-2.70) | 1.80(1.04-3.12) | 1.98(1.26-3.13) |
| Model 2 | 1.71(1.04-2.82) | 1 (ref.) | 1.49(0.83-2.65) | 1.70(0.95-3.06) | 1.76(1.07-2.89) |
| Model 3 | 1.39(1.10-1.76) | 1 (ref.) | 1.30(1.01-1.67) | 1.26(1.00-1.59) | 1.79(1.40-2.28) |

Data is weighted HR and 95%CI.

Model 1 is unadjusted model with additionally adjusted for age, age, sex, race, education level, annual family income, regular exercise habitus, smoking, drinking, BMI, sleep duration, daily energy intake, AHEI, meal skipping and whether dietary data was surveyed on weekend.

Model 2 is model 1 with additionally adjusted for fasting plasma glucose, TG, TC, drug use for controlling glucose/ hypertension/ dyslipidemia, family history of CVD.

Model 3 is model 2 with additionally adjusted for daily eating frequency.

Supplementary Table-9 The p-values for the modification effects of potential confounders on the association of daily eating frequency and nighttime fasting duration with mortality outcomes

| Confounders | Daily eating frequency | | | | Nighttime Fasting duration | | | |
| --- | --- | --- | --- | --- | --- | --- | --- | --- |
|  | All-cause | CVD | Heart-disease | Stroke | All-cause | CVD | Heart-disease | Stroke |
| Age | 0.142 | 0.650 | 0.175 | 0.159 | 0.445 | 0.415 | 0.58 | 0.609 |
| Sex | 0.085 | 0.164 | 0.08 | 0.159 | 0.809 | 0.187 | 0.3 | 0.609 |
| Smoke | 0.598 | 0.117 | 0.234 | 0.818 | 0.531 | 0.672 | 0.681 | 0.553 |
| Drink | 0.379 | 0.100 | 0.152 | 0.988 | 0.551 | 0.784 | 0.609 | 0.463 |
| Income | 0.266 | 0.341 | 0.051 | 0.646 | 0.313 | 0.055 | 0.893 | 0.412 |
| AHEI | <0.001 | <0.001 | 0.979 | 0.79 | 0.064 | 0.212 | 0.329 | 0.83 |

Data are P-values for the modification effects being different from 0.

Confounders in the weighted cox proportion hazards models included for age, age, sex, race, education level, annual family income, regular exercise habitus, smoking, drinking, BMI, sleep duration, daily energy intake, AHEI, meal skipping and whether dietary data was surveyed on weekend, fasting plasma glucose, TG, TC, drug use for controlling glucose/ hypertension/ dyslipidemia and family history of CVD.

Supplementary Table-10- Association of daily eating frequency with mortalities of all-cause and CVD among participants with AHEI below or over median

|  | Daily eating Frequency | | | | |
| --- | --- | --- | --- | --- | --- |
|  | Quintile 1  (< 3 times) | Quintile 2  (3 to 3.5 times) | Quintile 3  (3.5 to 4 times) | Quintile 4  (4 to 4.5 times) | Quintile 5  (4.5 to 6 times) |
| **AHEI** < **50** |  |  |  |  |  |
| All-cause mortality |  |  |  |  |  |
| Death/person-years | 770/61771 | 278/25276 | 334/28510 | 179/17433 | 292/29575 |
| Unadjusted | 1 .25(1.04-1.50) | 1.15 (0.93-1.43) | 1.19 (0.95-1.48) | 1.16 (0.92-1.45) | 1 (ref.) |
| Model 1 | 1.08 (0.89-1.31) | 1.00 (0.80-1.25) | 1.05 (0.83-1.33) | 1.04 (0.83-1.32) | 1 (ref.) |
| Model 2 | 0.95 (0.75-1.20) | 0.95 (0.75-1.20) | 0.98 (0.76-1.27) | 1.03 (0.81-1.31) | 1 (ref.) |
| Model 3 | 0.93 (0.73-1.18) | 0.96 (0.75-1.21) | 0.99 (0.77-1.27) | 1.04 (0.82-1.33) | 1 (ref.) |
| CVD mortality |  |  |  |  |  |
| Death/person-years | 296/61771 | 105/25276 | 126/28510 | 64/17433 | 109/29575 |
| Unadjusted | 1.42 (1.06-1.91) | 1.38 (0.95-2.01) | 1.33 (0.96-1.84) | 1.19 (0.80-1.77) | 1 (ref.) |
| Model 1 | 1.10 (0.81-1.49) | 1.09 (0.74-1.62) | 1.07 (0.77-1.49) | 1.01 (0.67-1.53) | 1 (ref.) |
| Model 2 | 0.94 (0.67-1.31) | 1.01 (0.70-1.47) | 0.98 (0.71-1.36) | 0.99 (0.66-1.48) | 1 (ref.) |
| Model 3 | 0.90 (0.63-1.30) | 1.00 (0.69-1.48) | 0.98 (0.70-1.38) | 0.99 (0.65-1.51) | 1 (ref.) |
| **AHEI > 50** |  |  |  |  |  |
| All-cause mortality |  |  |  |  |  |
| Death/person-years | 1109/46773 | 481/23960 | 455/25754 | 316/18412 | 343/28699 |
| Unadjusted | 2.24 (1.90-2.65) | 1.93 (1.62-2.31) | 1.60 (1.33-1.93) | 1.68 (1.42-1.99) | 1 (ref.) |
| Model 1 | 1.66 (1.41-1.95) | 1.56 (1.28-1.90) | 1.38 (1.12-1.69) | 1.39 (1.15-1.68) | 1 (ref.) |
| Model 2 | 1.46 (1.23-1.73) | 1.44 (1.19-1.75) | 1.29 (1.05-1.58) | 1.35 (1.12-1.63) | 1 (ref.) |
| Model 3 | 1.34 (1.13-1.60) | 1.38 (1.14-1.66) | 1.26 (1.02-1.55) | 1.34 (1.11-1.62) | 1 (ref.) |
| CVD mortality |  |  |  |  |  |
| Death/person-years | 501/46773 | 192/23960 | 193/25754 | 117/18412 | 120/28699 |
| Unadjusted | 3.05 (2.35-3.93) | 2.20 (1.67-2.89) | 2.13 (1.57-2.88) | 1.75 (1.30-2.34) | 1 (ref.) |
| Model 1 | 2.16 (1.65-2.83) | 1.72 (1.27-2.32) | 1.79 (1.30-2.45) | 1.41 (1.02-1.96) | 1 (ref.) |
| Model 2 | 1.76 (1.32-2.35) | 1.51 (1.11-2.06) | 1.61 (1.17-2.21) | 1.34 (0.97-1.86) | 1 (ref.) |
| Model 3 | 1.69 (1.25-2.29) | 1.49 (1.08-2.05) | 1.60 (1.16-2.20) | 1.35 (0.97-1.87) | 1 (ref.) |

Data is weighted HR and 95%CI.

Model 1 is unadjusted model with additionally adjusted for age, age, sex, race, education level, annual family income, regular exercise habitus, smoking, drinking, BMI, sleep duration, daily energy intake, AHEI, meal skipping and whether dietary data was surveyed on weekend.

Model 2 is model 1 with additionally adjusted for fasting plasma glucose, TG, TC, drug use for controlling glucose/ hypertension/ dyslipidemia, family history of CVD.

Model 3 is model 2 with additionally adjusted for daily eating frequency.
